# Development of a sandwich ELISA for the specific quantitation of hemagglutinin (HA)-tagged proteins during their inducible expression in *Escherichia coli*

**DOI:** 10.1007/s00216-023-04846-w

**Published:** 2023-07-28

**Authors:** Zihan Yin, Qiyi He, Huiyi Yang, Christophe Morisseau, El-Sayed A. El-Sheikh, Dongyang Li, Bruce D. Hammock

**Affiliations:** 1 Department of Entomology and Nematology, and UCD Comprehensive Cancer Center, University of California, Davis, CA 95616 USA; 2 Plant Protection Department, Faculty of Agriculture, Zagazig University, Zagazig, 44511 Egypt; 3 Laboratory of Agricultural Information Intelligent Sensing, College of Biosystems Engineering and Food Science, Zhejiang University, Hangzhou, 310058 Zhejiang China

**Keywords:** Nanobody, VHH, Protein expression, Sandwich ELISA

## Abstract

Heavy single-chain antibodies (VHH or nanobodies) are popular in the medical and analytical fields due to its small size, high solubility, stability, and other advantageous features. However, the usage of VHHs is limited by the low yield of its production and purification. In order to determine the optimal purification strategy for VHH to improve the yield, a method to monitor purification at the intermediate steps is needed. In this study, a simple, sensitive, low-cost sandwich enzyme-linked immunosorbent assay (ELISA) was developed to quantitate VHHs throughout the purification steps. Under optimized conditions, the assay has a sensitivity of 0.149 OD·mL/ng and a limit of detection (LOD) of 0.029 ng/mL. The average recoveries of the assay against the spiked samples were 101.9–106.0% and 100.7–108.0%. The method was applied to a variety of real samples for the detection of different VHHs in bacterial cell media. High amount of VHHs (up to 41.3 mg/mL), which are comparable to the average yield of VHH in standard production protocols, were detected in the media. This study raises attention to the problem of protein losses in cell culture supernatants and provides a method for the continuous detection of the protein abundance to optimize the expression and purification protocols especially for nanobodies.

**Graphical abstract:**

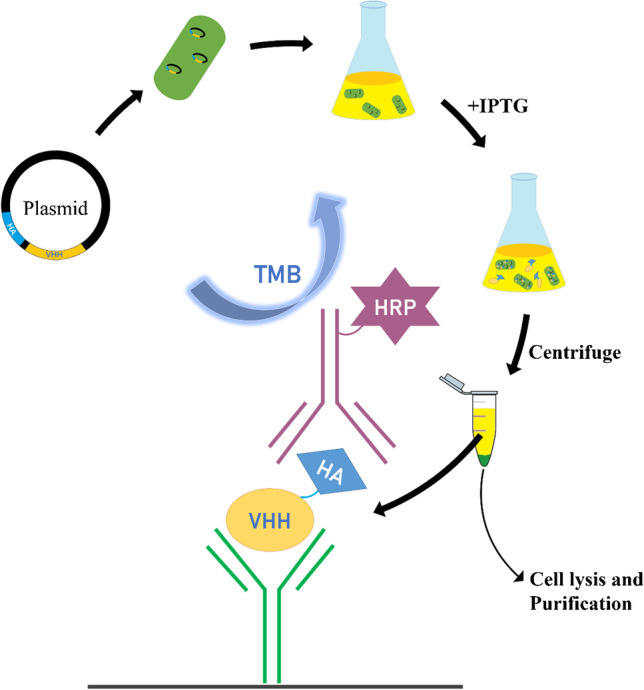

**Supplementary Information:**

The online version contains supplementary material available at 10.1007/s00216-023-04846-w.

## Introduction

*Escherichia coli* has become a popular expression system over the past 50 years. Features such as rapid growth rate at high density, small, well-characterized, and easily modified genome, as well as the availability of numerous mutant strains at low cost make *E. coli* a great host for heterogeneous protein expression [1]. A typical protocol of cytoplasmic protein expression involves a series of separation/purification steps. As the first step of this procedure, centrifugal fractionation is often assumed effective enough to perfectly separate the cells that should contain all of the expressed protein from the used media. However, because of cell death and leakage into the media, the supernatant always contains a proportion of the target protein [2]. When the protein has an enzyme activity, it is straight-forward to test both cell pellet and supernatant to determine the optimal time to collect the cells to maximize the amount of targeted protein in the pellet. On the other hand, it becomes very difficult to optimize the recombinant protein production when the protein does not possess an enzymatic activity, such as antibodies. In this case, the protein yield is usually measured at the terminal step of protein purification for simplicity. The expression conditions and purification methods that give higher yield are considered more efficient. A limitation of this criterion is that one cannot distinguish if the higher yield is from good expression or high purification recovery. There is a good chance that the overall production of the purified protein is less than optimal and that a sizeable amount of protein could be lost along the way, underlying the need to quantify the targeted protein at every step of the way for better recovery. Moreover, the optimized purification protocols would reduce the proportion of the protein loss in the cell media, but does not guarantee a low quantity of protein lost. Alternatively, the target protein can be retrieved from the cell media by additional purification steps when necessary. However, the currently available methods to extract specific target proteins from cell media are complicated and sometimes costly. Thus, it would be efficient in terms of time and cost to estimate the abundance of target protein in the cell media before undergoing further purification with a simple and low-cost quantitative method.

The accuracy of traditional colorimetric methods for protein quantitation (e.g., UV absorbance (A280/A260), Bradford assay, BCA assay) are limited by the purity of protein samples because they quantify the total amount of proteins rather than a specific target protein [3–7]. In this decade, the advancement of technology has made mass spectrometry (MS) a useful tool for not only protein identification but also protein quantification, especially in proteomic analysis. Common MS-based methods such as MS, MS/MS, and LC–MS/MS are able to quantify proteins or peptides with good sensitivity and reproducibility [8, 9]. But the quantitative sensitivity of MS-based methods is typically lower than that of antibody-based methods. Moreover, these methods are limited by the requirement of complicated pretreatment of samples, high costs of instrument, and skilled operator. None of the methods above could satisfy the need to monitor the intermediate steps of preparation for more comprehensive evaluation of the purification protocols. Hence, developing a simple, effective, and low-cost protein quantification method in the cellular matrix is needed.

Accurate quantitation of a single protein in complex cellular matrix requires highly specific and selective recognition between the analyte and the reporter. A classic example of such interaction is the exclusive interactions between an antigen and a specific antibody. Enzyme-linked immunosorbent assays (ELISA) are highly sensitive, simple, low cost, and high-throughput analytic methods that have wide applications on the detections of a myriad of analytes [10].

For the past two decades, the bioanalytical field has been transformed by the usage of recombinant antibodies, especially nanobodies (heavy single-chain antibody; VHH). With a small size of ~ 15 kDa, VHH has comparable or even more advantageous properties over the conventional polyclonal or monoclonal antibodies. Besides, VHHs are now widely used in diagnostic, therapeutic, and analytic fields because of their high specificity, solubility, thermostability, proteolytic resistance, ease of genetic manipulation, storage, expression, and many other key features [11, 12]. While VHH could be easily recombinantly produced in *E. coli*, a limitation to their usage is variation and low yield in VHH production and purification.

Toward solving this problem, an ELISA method to detect multiple VHHs was developed. The majority of the published and commercialized VHH sequences contain a HA (hemagglutinin) tag as an extensive epitope for the purpose of isolation, purification, and detection [13]. The developed sandwich ELISA for VHH detection took advantage of the HA tag using commercial anti-VHH polyclonal antibody (pAb) and horseradish-peroxidase-labeled anti-HA tag monoclonal antibody (mAb) conjugate (HRP-anti-HA tag). To the best of our knowledge, there has no reported ELISA targeting the HA tag to quantify VHH in cellular media. Only one research paper found has reported an indirect ELISA to quantify the anti-rabies VHH, but this method does not meet the need to assess the purification efficiency of VHH [14]. First, the ELISA was not optimized to reach the best performance for general use. Further, this method was applied to plasma rather than cell media, which could have different matrix effects. More critically, the ELISA was specific to single type of VHH. However, developing an ELISA for each recombinant protein could be costly. Thus, a method that can specific ally recognize a class of recombinant protein will be cost effective in optimizing their production. Our ELISA-based method developed in this study can be easily used to determine the following purification strategy and can potentially be used to better assess the expression efficiency and optimize expression conditions of a variety of VHHs.

## Material and methods

### Materials

The production of anti-human soluble epoxide hydrolase (human sEH) VHHs (A1, A9, B13) was described in our previous work [15]. The anti-3-phenoxybenzoic acid (3-PBA) VHH/3P5ThC12, anti-mouse soluble epoxide hydrolase (mouse sEH) VHH/4C3, and anti-aflatoxin B1 (AFB1) VHH/2–5 used in the experiments were produced in the lab [10, 16, 17]. Anti-VHH rabbit pAb was prepared using similar procedure as described in Lee et al. by the injection of anti-human sEH VHH A9 into New Zealand white rabbits [18]. Antisera were purified by protein-A-agarose affinity chromatography. The purified antibodies were obtained via dialysis in phosphate buffered saline (PBS). High Affinity Anti-HA-Peroxidase, clone 3F10 monoclonal antibody (HRP-anti-HA tag), was purchased from Roche. The preparation of 3,3′,5,5′-tetramethylbenzidine (TMB) substrate for color development is described in the previous publication [19]. Bovine serum albumin (BSA) (Lot: BP1600-100) and skim milk (SM) powder (Lot: 1.15363.0500) were purchased from Thermo Fisher Scientific and EMD Millipore, respectively. Nunc MaxiSorp high-binding flat-bottom 96-well plates were purchased from Thermo Fisher Scientific. The 96-well plates were automatically washed on a 96-channel plate washer AquaMax 2000 (Molecular Devices). The optical density (OD) of microwells were recorded by a microplate reader SpectraMax 190 (Molecular Devices) at single wavelength of 450 nm.

### Sandwich ELISA for VHH detection

**Fig. 1:**
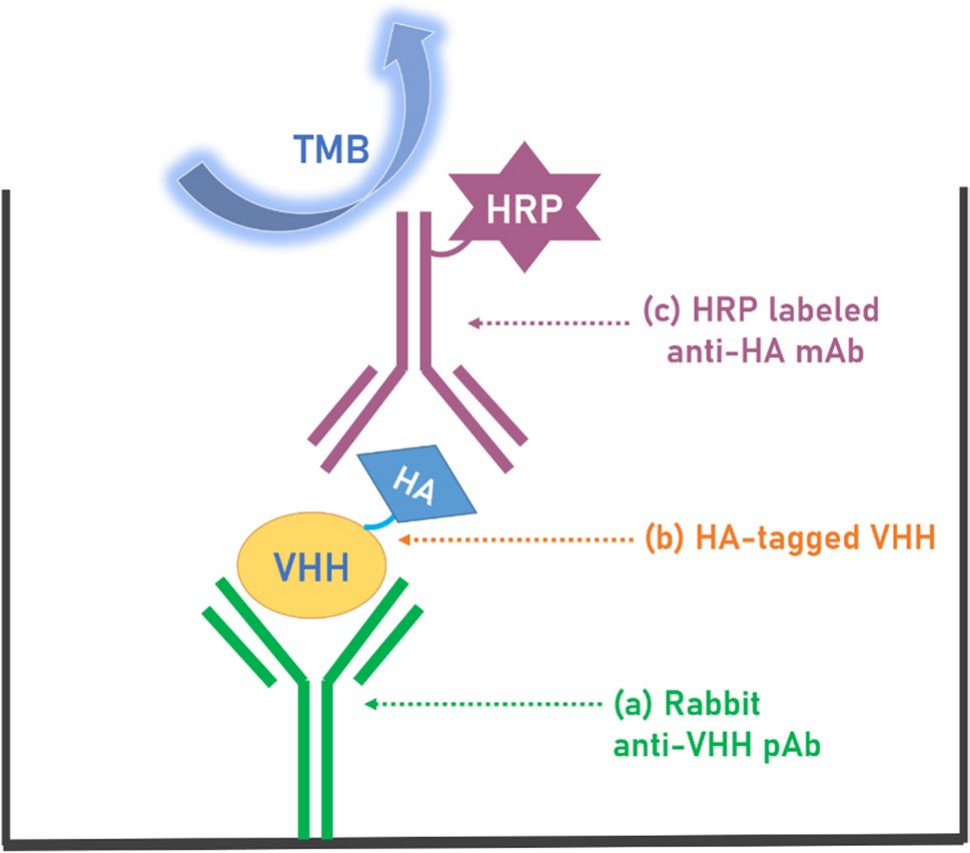
Illustration of the sandwich ELISA scheme for VHH detection, from bottom to top: (**a**) rabbit anti-VHH pAb which was passively absorbed at the bottom of the well as a capture antibody (**b**) analyte (**c**) HRP-anti-HA tag mAb as a detection antibody

The scheme of the assay is demonstrated in Fig. 1. The 96-well microtiter plate was coated overnight at 4 °C with purified rabbit polyclonal antibody in 0.05 M, pH 9.6 carbonate–bicarbonate buffer (100 μL/well). On the next day, the uncoated sites on the plate were blocked with 3% (w/v) skim milk (225 μL /well) in PBS for 1 h and followed by washing. Serial dilutions of the VHH standard in PBS were added to the wells (100 μL/well) for 1 h immunoreaction. The microplate was washed again prior to the addition of HRP-anti-HA tag mAb in PBS (100 μL/well). The final five washings ensured the thoroughly removal of the unbound antibodies and then freshly prepared 3,3′,5,5′-tetramethylbenzidine (TMB) substrate solution (100 μL/well) which was added to wells for a 15-min incubation. The optical density (OD) was recorded at 450 nm as soon as the color development reaction was terminated by adding 1 M sulfuric acid (100 μL/well). All incubations were conducted at room temperature (RT) with shaking (600 rpm) on the MTS 2/4 digital microtiter shaker of 4-plate capacity (IKA) unless otherwise specified. Each washing step involved three washings with PBS containing 0.05% Tween-20 (PBST, 300 μL/well) using the plate washer.

### Optimization of sandwich ELISA

The optimal conditions of the sandwich ELISA, including coating concentration, secondary antibody concentration, and blocking reagent, were determined independently using a representative VHH (A1) as standard. The immunoassay was firstly run at five levels of the capture antibody (1, 2, 4, 6, 8 μg/mL) to determine the optimal coating concentration. The range of concentrations of A1 was extended until the absorbance measurements no longer exhibit linearity (0–100 ng/mL). Another set of plate were coated with the optimal coating level (4 μg/mL), and twofold serial dilutions of the HRP-anti-HA tag (16.7, 8.33, 4.17, 2.08, 1.04, 0.52, 0.26, 0.13 ng/mL) were added over 0–50 ng/mL of A1. Furthermore, the blocking efficiency using bovine serum albumin (BSA, 2%) or skim milk (SM, 3%) was compared over four concentrations of capture antibody using 8.33 ng/mL HRP anti-HA tag for detection. The rest of the conditions were kept the same as above. All the individual factors of ELISA were tested simultaneously, if not on the same plate, to minimize variations and improve precision of the results.

### Cross-reactivity

A total of seven biomolecules were tested for cross-reactivity with VHHs A1 (anti-human sEH) using the sandwich ELISA, which included four VHHs and three non-immunoproteins. Among the four VHHs, VHH B13 (anti-human sEH) and 4C3 (anti-mouse sEH) were obtained against large biomolecular targets, while VHH 3P5ThC12v (anti-3-PBA) and VHH 2–5 (anti-AFB1) were against small molecular targets. The three tested proteins (BSA, yeast extract, and tryptone) are commonly used in ELISA and in cell media. The assay was set up under the optimal condition (4.0 μg/mL capture pAb, 8.33 ng/mL HRP anti-HA tag), and the tested compounds were serially diluted (0–20 ng/mL). The cross-reactivity of the ELISA was calculated as follows:

1 $$Cross\,Reactivity \left(\%\right)=\left[\frac{{k}_{protein}}{{k}_{VHH A1}}\right]\times 100\%$$

in which k(i) is the sensitivity (slope of the fitted linear curve) of the biomolecule in the sandwich ELISA.

### Spike-and-recovery test

The accuracy and precision of the sandwich ELISA were evaluated by spiking the blank cell media sample dilutions with serial dilutions of VHH A1. To prepare the blank samples, the pComb3X plasmids containing no protein insert were transformed into TOP10F’ cells by heat shock. The expression procedures were the same as previously described and can be found in the Supplemental Information [17]. The blank cell media samples were obtained 2 h after IPTG induction and after overnight induction. The collected samples were centrifuged for 30 min, and the supernatants were used for analysis. Briefly, the supernatant samples were diluted by 1000-, 5000-, and 10,000-fold with PBS. The three sample dilutions were then spiked with serial dilutions of VHH A1 (0–20 ng/mL) and analyzed by the sandwich ELISA method described above.

### Real sample analysis

The pComb3X plasmids containing VHHs A1, 4C3 and 3P5ThC12v were transformed into TOP10F’ cells by heat shock separately. The procedures for the expression of VHHs and sample collection were the same as in the spike-and-recovery test. The supernatants were sampled 2 h, 6 h, and overnight after the addition of IPTG. The samples were serially diluted using PBS and analyzed by the sandwich ELISA to quantify their VHH content. Sample dilution(s) which had the measured OD falling into the linear range of the calibration curve was/were used to calculate the VHH concentrations in the samples.

## Results and discussion

### Parameters used for evaluating assay performance

The lack of a standardized criteria for systematic assessment of ELISA performance often leads to obscurity and inconsistency of statistical values used for evaluation. In order to identify the optimal conditions for an ELISA, we should first define what ‘optimal’ means. Accuracy and precision are the two terms commonly used to evaluate a method. The former term represents the closeness to the true value, while the latter is defined as the reproducibility of the data. In this study, the two terms were featured by the mean, standard deviation (SD), and coefficient of variation (CV) of the data.

Meanwhile, additional criteria should be considered when evaluating a ELISA method, such as sensitivity, limit of detection (LOD), and range of linearity. Ambiguities are often found in these quantities as well. For sandwich ELISA, the common approach is to identify the range of the dose–response curve that is approximately linear. The general expression of the fitted linear curve is

2 $$y=kx+b$$

where *x* is the concentration of analyte, *y* is the optical density of the sample, and *k* and *b* are the slope and intercept of the fitted line, respectively. The linearity of the fitted curve is assessed by the *R*^2^ value, which suggests that the does-response trend is linear when the value is closer to 1. The range of analyte concentration with *R*^2^ value > 0.99 is considered as the linear range of the calibration curve. Here, we adapted the formal definition of sensitivity, which is the rate of change of the response (dR) to a given change in stimulus (dC). Therefore, mathematical expression for the sensitivity (S) can be as follows:

3 $$S=\frac{dR}{dC}$$

Throughout this study, the reported sensitivity of an ELISA upon testing a compound is the slope of the fitted linear curve (*k*) on the dose–response plot of that compound using least squares method in linear regression. The resulted value represents the absorbance of the sandwich antibody complex per ng VHH, with unit of OD·mL/ng.

By the classic definitions of these parameters, the sensitivity is distinguished from the limit of detection (LOD). LOD is the smallest amount of analyte that can be resolved with a given degree of confidence and can be computed as

4 $$LOD= 3\frac{{s}_{b}}{k}$$

where *k* is the slope or sensitivity of assay against analytes and *s*_*b*_ is the sample standard deviation of the blank (*n* = 3) [20].

Despite the use of well-defined assessment quantities, the assay performance is affected by numerous factors, such as room temperature fluctuation, incubation time, pipetting, and numbers of replicates, which increases the difficulty for inter-assay comparison [21]. In order to minimize the impact of environmental factors, the conditions of the assay were strictly controlled. Data used for comparison were collected in single run and on the same plate if possible. Computations of the statistics and visualization of the data were accomplished using OriginPro 8.5 SE for this study.

### Optimization of sandwich ELISA

The VHH A1 and A9 are two anti-human sEH VHHs screened under the same conditions from a common phage library in the previous study [17]. The sequence identity and similarity between A1 and A9 are as high as 67.5% and 85.5% (Smith-Waterman scoring algorithm was used for sequence alignment), which ensures similar structures and activities [22]. Thus, the signals caused by A1 was expected to be similar to that by A9 in the assay. Based on these evidences, A1 was selected to construct calibration curve in assay optimization and sample analysis due to the limited supply of the immunogen A9.

**Fig. 2:**
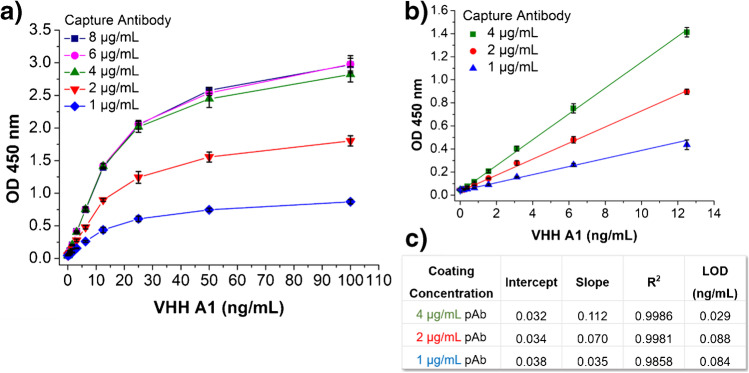
**a** Signal responses of sandwich ELISA using five concentrations (1, 2, 4, 6, 8 μg/mL) of capture antibody; **b** linear portion of the signal responses at 1 μg/mL (▲), 2 μg/mL (●), and 4 μg/mL (■) of capture antibody; and **c** parameters of the linear regression of signal responses at 1 μg/mL (▲), 2 μg/mL (●), and 4 μg/mL (■) of capture antibody passively absorbed on high-binding polystyrene microplate. HRP-anti-HA tag (1:3000 dilution or 8.33 ng/ml) was used as the detection antibody, and 3% SM was used for blocking step. Error bars indicate standard deviations (*n* = 3). All coefficients of determination *R*^2^ > 0.98

Figure 2 shows the signal-response curves of the sandwich ELISA against A1 using five concentrations of capture antibody. As shown in Fig. 2a, the signal intensity (i.e., OD) increases with the increasing of capture antibody from 1 to 4 μg/mL. The curves showed no significant difference when the concentration of capture antibody reached above 4 μg/mL. Besides, the range of linearity remained the same over the varying concentrations of the capture antibody at 0.19–12.5 ng/mL. Therefore, the data of coating concentrations below 4 μg/mL were examined more closely in Fig. 2b and c. The sensitivity (slope) of the assay increased in proportion to the increase of coating concentration, while the blank signal did not show significant change. Hence, 4 μg/mL capture antibody was used for further testing to minimize the LOD and achieve a relatively high sensitivity.

**Fig. 3:**
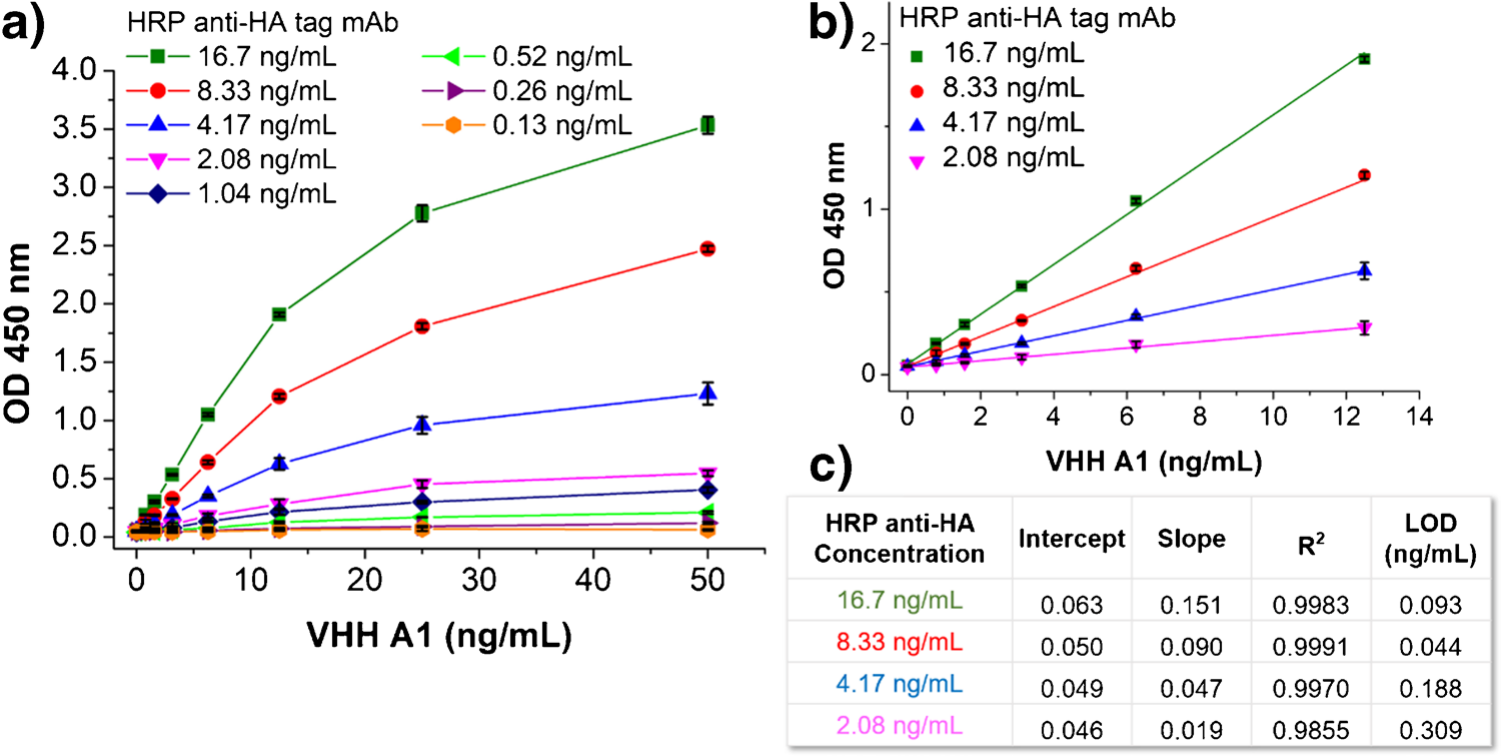
Signal responses of sandwich ELISA using 4 μg/mL of capture antibody passively absorbed on high-binding polystyrene microplate followed by 3% skim milk (SM) as blocker. **a** Eight concentrations (0.13–16.7 ng/mL) of HRP-anti-HA tag were used as detection antibodies. **b** Linear portion of the signal responses using 2.08–16.7 ng/mL of detection antibody. **c** Parameters of the linear regression of signal responses using 2.08–16.7 ng/mL of detection antibody. Error bars indicate standard deviations (*n* = 3). All coefficients of determination *R*.^2^ > 0.98

Similarly, a series of concentration (16.7, 8.33, 4.17, 2.08, 1.04, 0.52, 0.26, 0.13 ng/mL) of detection antibody was tested and the curves are illustrated in Fig. 3. As expected, the detection signal increased with the increasing concentration of detection antibody. When the concentration of detection antibody was below 2.08 ng/mL, the sensitivity of the assay was too low to measure the concentration of analyte. To compare the performance of the assay at the highest four concentrations (16.7–2.08 ng/mL) of HRP anti-HA with more details, the results of linear regression are shown in Fig. 3b and c. While the increase of the concentration of detection antibody from 8.33 to 16.7 ng/mL improved the sensitivity of the assay by 167%, it also led to an increase of 214% in LOD due to higher background. Since the sensitivity and LOD increased together, the overall improvement is very little. Therefore, we chose the lower concentration 8.33 ng/mL for less material consumption. Thus, 8.33 ng/mL (1:3000 dilution of the commercial product) was selected among the eight tested dilutions as the optimal concentration of HRP anti-HA mAb.

**Fig. 4:**
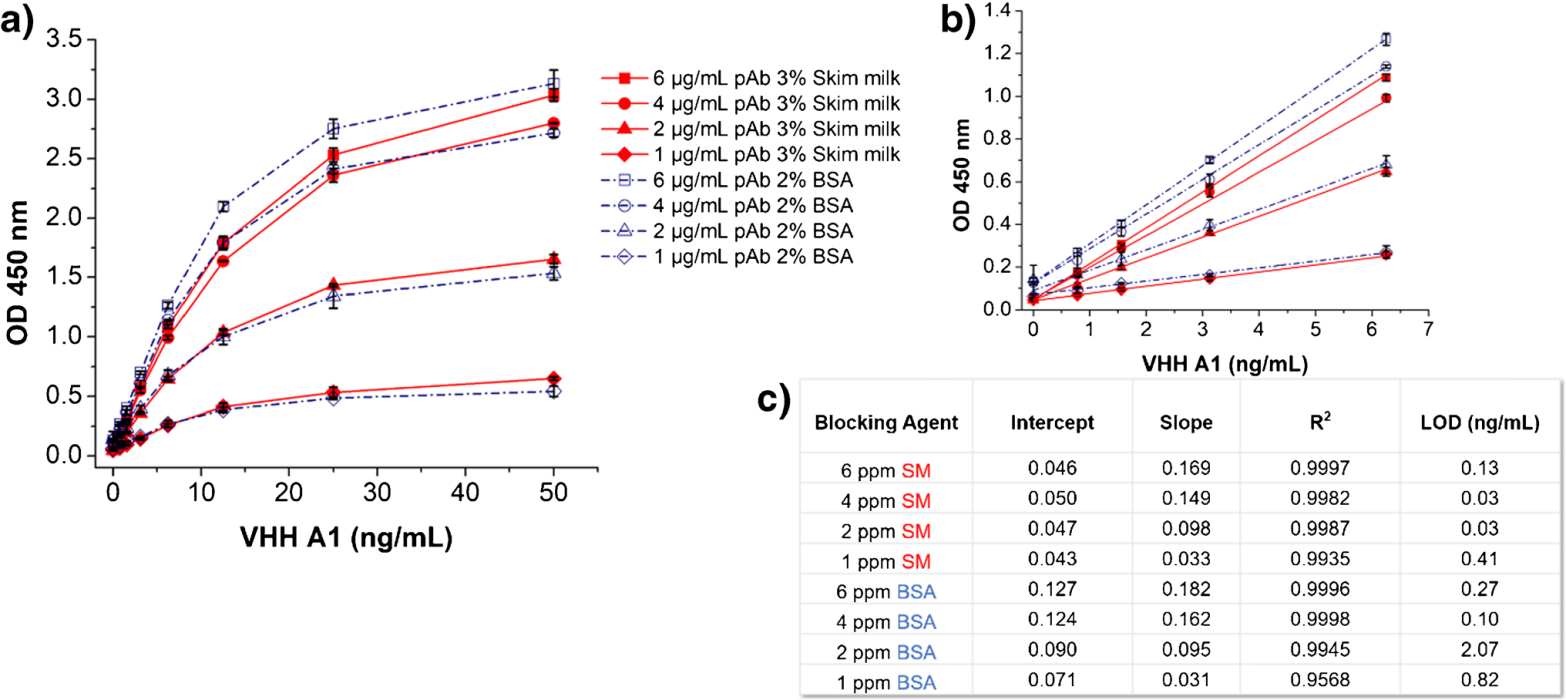
**a** Signal responses, **b** linear portion, and **c** parameters of the linear regression of the signal responses of sandwich ELISA using 1 μg/mL (♦), 2 μg/mL (▲), 4 μg/mL (●), and 6 μg/mL (■) of capture antibody passively absorbed on high-binding polystyrene microplate then blocked by either 3% skim milk (solid) or 2% BSA (hollow). HRP-anti-HA tag (1:3000 dilution or 8.33 ng/ml) was used as detection antibodies. Error bars indicate standard deviations (*n* = 3). All coefficients of determination *R*^2^ > 0.95.

Another interfering factor to the performance of ELISA is nonspecific binding (NSB) with the plastic or unoccupied active surface of the antibodies. Minimizing NSB prevents false-positive signals and improves the signal-to-noise ratio, therefore leading to higher sensitivity of the assay. Previous study has proved that skim milk and BSA are both effective blocking agents [23]. Skim milk is commonly used due to its similar or often better blocking effectiveness than BSA, lower expenses, availability, stability, and simple storage method [24]. We compared the two blocking agents at their commonly used concentrations, 2% BSA and 3% skim milk in PBS, over four dilutions of capture antibodies to determine their power of blocking NSB empirically. The signal intensity (Fig. 4a) of the entire curves exhibited no significant difference between the two blocking agents. However, after closer examination of the data (Fig. 4b), it can be seen that BSA blocking resulted in higher background signal, which resulted an increase in LOD by two fold (Fig. 4c). Therefore, skim milk (3%) was used in the optimized method.

Under the optimal conditions, the assay gave the lowest LOD of 0.029 ng/mL and the highest sensitivity of 0.149 OD·mL/ng (Fig. 4c and Table S-1). The precision of the assay was evaluated by plate-to-plate variation at this stage. The averages and CV were obtained using the data collected for the assay run under the optimal conditions during the optimization process, shown in Figs. 2, 3, and 4 (also described in Supporting Information). The plate-to-plate average (CV) of sensitivity and LOD were 0.117 OD·mL/ng (25.3%) and 0.036 ng/mL (20.8%). The reproducibility of the assay was acceptable. The further evaluation of the accuracy and precision of the assay is reported in the following section.

### Method validation

In order to assess the accuracy of the method, the spike-and-recovery test was performed using spiked blank cell media supernatant at a series of dilutions. Blank supernatant samples were collected after 2 h and overnight IPTG induction, which were then spiked with serial dilutions of A1. The results of the tests are listed in Table 1. No apparent change in background signal was observed in the supernatants at either the initial (2 h) or terminal (overnight) of IPTG induction process. Increasing the dilution factor to 1:10,000 did not cause a significant difference in signal responses against the supernatant after induction. The average recoveries of the spiked samples after 2 h and overnight IPTG inductions were 101.9–106.0% and 100.7–108.0%, respectively. The CV was kept below 12.5% for all the spiked samples.

**Table 1:** Recoveries of pure VHH A1 spiked into the 2-h and overnight IPTG-induced supernatant samples with different dilution factors (*n* = 3)

	2-h induction^*^								
	1:1000 dilution		1:5000 dilution				1:10,000 dilution		
Spiked (ng/mL)	Detected (ng/mL)	Recovery (%)	Detected (ng/mL)		Recovery (%)		Detected (ng/mL)		Recovery (%)
0.00	0.16 ± 0.01 (6.93%)	-	0.11 ± 0.01 (5.92%)		-		0.10 ± 0.01 (12.49%)		-
0.78	0.84 ± 0.02 (2.20%)	107.0	0.79 ± 0.01 (1.71%)		101.6		0.84 ± 0.07 (8.25%)		107.9
1.56	1.63 ± 0.05 (3.37%)	104.2	1.51 ± 0.01 (0.98%)		96.7		1.59 ± 0.02 (1.04%)		101.5
3.13	3.62 ± 0.05 (1.51%)	115.9	3.39 ± 0.05 (1.36%)		108.4		3.45 ± 0.13 (3.80%)		110.4
6.25	6.59 ± 0.38 (5.81%)	105.5	6.49 ± 0.24 (3.64%)		103.9		6.49 ± 0.15 (2.31%)		103.8
12.50	12.21 ± 0.68 (5.58%)	97.7	12.37 ± 0.04 (0.28%)		99.0		12.31 ± 0.32 (2.63%)		98.5
Average recovery (%)	-	106.0	-		101.9		-		104.4
	Overnight induction^*^								
	1:1000 dilution		1:5000 dilution			1:10,000 dilution			
Spiked (ng/mL)	Detected (ng/mL)	Recovery (%)	Detected (ng/mL)	Recovery (%)		Detected (ng/mL)		Recovery (%)	
0.00	0.13 ± 0.003 (2.42%)	-	0.10 ± 0.004 (3.62%)	-		0.13 ± 0.002 (1.42%)		-	
0.78	0.81 ± 0.09 (10.96%)	103.6	0.77 ± 0.01 (1.71%)	98.1		0.84 ± 0.096 (11.38%)		107.9	
1.56	1.54 ± 0.09 (5.91%)	98.6	1.56 ± 0.008 (0.49%)	99.7		1.60 ± 0.03 (2.01%)		102.5	
3.13	3.25 ± 0.11 (3.50%)	104.0	3.35 ± 0.07 (2.19%)	107.2		3.69 ± 0.21 (5.80%)		118.2	
6.25	6.43 ± 0.28 (4.43%)	102.9	6.42 ± 0.23 (3.62%)	102.7		6.93 ± 0.04 (0.64%)		110.8	
12.50	12.09 ± 0.37 (3.02%)	96.7	11.99 ± 0.35 (2.94%)	95.9		12.55 ± 0.21 (1.68%)		100.4	
Average recovery (%)	-	101.2	-	100.7		-		108.0	

^*^Results are average ± SD (CV), *n* = 3

The fluctuation (CV) was below 5% for spikes above 1.56 ng/mL, indicating a high precision of the assay. Overall, the results of the spike-and-recovery and sensitivity test demonstrated the satisfactory accuracy, precision, and sensitivity of the method in blank samples, supporting the applicability of the sandwich ELISA for VHH detection in the supernatant after induction.

### Performance of sandwich ELISA

**Fig. 5:**
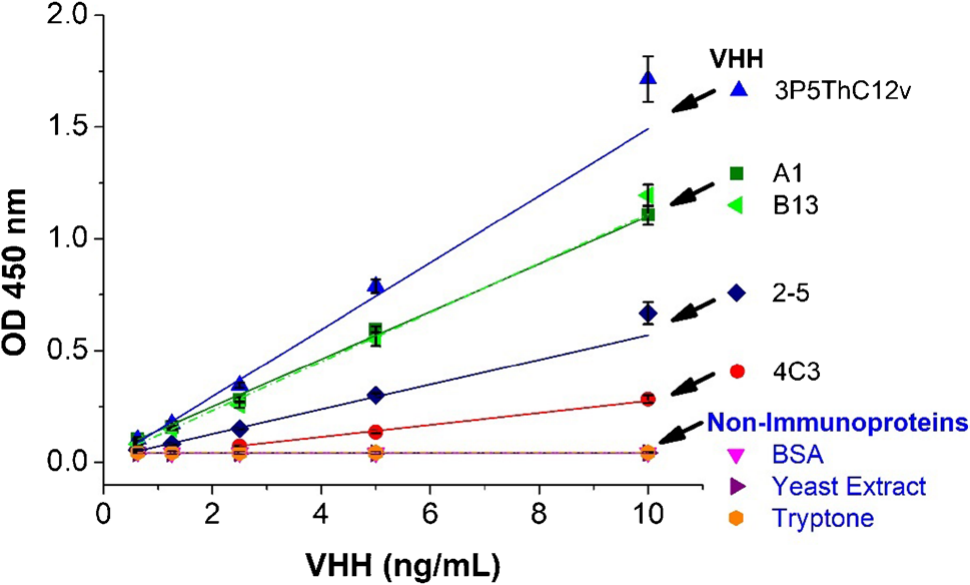
Signal responses of sandwich ELISA applied against five different VHHs and three non-immunoproteins in the range of 0.31–10 ng/mL. Capture antibody (4 μg/mL) was passively absorbed on high-binding polystyrene microplate, and 3% SM was used for blocking. HRP-anti-HA tag (1:3000 dilution or 8.33 ng/ml) was used as detection antibody. Error bars indicate standard deviations (*n* = 3)

The design of the sandwich ELISA is aimed to be applicable to a variety of VHHs based on the similarity in the sequence and structure of VHH. An ideal method would exhibit high cross-reactivity against VHHs and a low cross-reactivity against non-VHH molecules. The cross-reactivity was evaluated by the ratio of the slope of the signal-response curves (slope of analyte/slope of A1). The results of the cross-reactivity test of the pAb-based sandwich ELISA against various VHHs are shown in Fig. 5 and Table 2. The assay showed high cross-reactivity of 103% against B13, which was the anti-human sEH VHH obtained using the same screening method as A1. The assay was also highly sensitive to anti-3-PBA VHH 3P5ThC12v with the cross-reactivity of 140%. The activity against anti-aflatoxin B1 (AFB1) VHH 2–5 and anti-mouse sEH VHH 4C3 were 52% and 25%, respectively. Despite the sequence identity of 70–80% between each tested VHH and A1, not all the VHHs were equally recognizable by the assay. This variation can be attributed to the nature of pAb that recognize multiple sites of the antigen. The pAb tends to be more specific to the immunogen and exhibits different cross-reactivity to the antigens with similar structures. In addition, VHH A1 and B13 are llama sourced, whereas 3P5ThC12v and 2–5 are alpaca sourced, which could also contribute to the difference in CR. Although the activity against some VHHs was lower than 70%, the assay was sensitive enough to generate OD ~ 1.0 at trace level of the VHHs of 10 ng/mL. It means that the assay is still applicable with acceptable sensitivity to detect VHHs with low CR with A1. Evaluation of VHH abundance could be done by simply using the same VHH analyte or VHH with higher sequence identity to generate the standard curve. For the three tested proteins, the results showed negligible cross-reactivity against interferants such as the proteins in cell medium (i.e., yeast extract and tryptone) or BSA from the blocking steps. The data further proved the general applicability of the assay for the detection of different VHHs.

**Table 2:** Cross-reactivity of sandwich ELISA for selected VHHs and non-immunoproteins

VHH	VHH Targets	Cross reactivity (%)	Non-immunoproteins	Cross reactivity (%)
A1	human sEH	100	BSA	< 0.1
B13	human sEH	103	Yeast extract	< 0.1
3P5ThC12v	3-PBA	141	Tryptone	< 0.1
2–5	AFB1	52		
4C3	mouse sEH	25		

### Real sample analysis by ELISA

The applicability of the established ELISA method in analysis of real samples was illustrated in this study. Samples of cell media were collected 2 h, 6 h, and overnight after induction of *E. coli* by IPTG during the production of VHHs A1, 4C3, and 3P5ThC12v. The three VHHs were selected based on their acceptable cross-reactivity and the availability of plasmid in the lab. A calibration curve was constructed using A1 with a good fitness for linearity (*R*^2^ > 0.999). The concentrations of VHHs calculated using the calibration curve are given in Table 3. The optimal dilutions for the VHH A1, 4C3, and 3P5ThC12v supernatant samples tested by the ELISA were 1000-, 100-, and 1000-fold for both 2-h and 6-h IPTG induction and 10,000-, 100-, and 10,000-fold for overnight IPTG induction, respectively. The sandwich ELISA successfully detected more than 40 mg/mL A1 and 13 mg/mL 3P5ThC12v in the supernatant after overnight induction. The detected concentrations of A1 and 3P5ThC12v were highly consistent with the CV ranged from 0.2 to 6%. The measured concentration of 4C3 after 2-h induction showed a high fluctuation that may due to the low responses of the assay against 4C3 and the low abundance of 4C3 in the supernatant. The evidence that supports this explanation is that the precision of the assay was largely improved in the samples of 4C3 after overnight induction. Amplification of VHH abundance in the supernatants from 2 h to overnight induction was observed across all three VHHs. The concentrations of A1, 4C3, and 3P5ThC12v increased by 23-, 3-, and 50-fold during the induction process, respectively.

**Table 3:** Detected VHH by sandwich ELISA in supernatant samples after 2-h, 6-h, and overnight IPTG induction during the expression of the three VHHs

	Detected by ELISA (mg/ml)^*^		
VHH	2 h	6 h	Overnight
A1	2.87 ± 0.10 (4%)	3.58 ± 0.06 (1%)	41.28 ± 1.74 (4%)
4C3	0.07 ± 0.02 (27%)	0.19 ± 0.08 (7%)	0.21 ± 0.003 (1%)
3P5ThC12v	0.27 ± 0.01 (2%)	1.99 ± 0.05 (1.0%)	13.63 ± 0.03 (0.2%)

^*^Results are presented as mean ± SD (CV), *n* = 3

Due to the lack of quantification methods, no reported data on the VHH abundance in the supernatants has been found. For the same reason, it is also difficult to compare and confirm the measurement in this study. Hence, our measurements gave the data of VHH abundance in cell media supernatants for the first time. The concentration of the detected VHHs in the supernatants, especially for A1 and 3P5ThC12v, is high enough to raise our attention since a yield at mg/L level is considered high for VHH production, indicating that it is worthwhile to retrieve the protein in the following purification protocols [2, 12]. The retrieval of the lost VHH in the purification process can elevate the protein production by exponential fold, although one of the challenges to recover such large amount of VHH in the supernatant after induction is the cellular debris and interferent proteins that severely affect the efficiency of affinity chromatography. Two studies that attempted to extract proteins from cell culture supernatant have been found. Rosa et al. successfully purified IgG from Chinese hamster ovary cell culture supernatant by an aqueous two-phase extraction (ATPE) process based on a PEG/phosphate system [25]. The global recovery yield using this extraction method reaches 80% with the final purity of the protein over 99%. Despite of its high capacity, the ATPE method requires complicated pump-mixer operating counter, involves repetitive extraction procedures, and lacks the ability of direct quantitation of the extracted proteins prior to purification. More recently, Liu et al. developed a nano-immunoaffinity platform using Protein A- and Protein G-gold nanoparticle bioconjugates for quantitative antibody extraction from OKT-8 cell culture supernatant in monoclonal antibody production [26]. The bioconjugate platform allows efficient protein extraction by simply pipetting nanoparticle solutions and a mini-spin. There is one problem with this method that the bioconjugates are not available for purchase and must be synthesized in lab. Both of these methods require complicated preparation for the extraction in return of the high recovery. Estimation of the protein abundance in the cell culture supernatant using the developed sandwich ELISA can give a reference to decide if the supernatant would undergo additional purification steps for protein recovery. This method can also be applied to a different matrix, more specifically, at every stage of protein purification, in the future. Optimizations and adjustments are required to eliminate the matrix effect by each matrix, for example, the cell lysate. One possible limitation of this method is the cross-reactivity among the VHHs despite high sequence identity. At this stage, it can be solved by selecting the standard VHH. However, if one wants to apply the assay to multiple VHHs at the same time, the cross-reactivity may be checked before quantification. Improvement of sensitivity by further amplifying the signal responses can also compensate the low CR between the tested VHH and the standard VHH. Another potential application of the assay is to predict the protein content inside the cell after induction without cell lysis. In order to do so, the correlation between the protein abundance inside the cell and in the media should be studied in the future.

## Conclusions

A sensitive and specific sandwich ELISA for VHH detection in cell media using commercially available rabbit anti-VHH pAb as capture antibody and HRP labeled anti-HA tag mAb as detection antibody was developed. Under the optimized conditions, the assay gives the highest sensitivity of 0.149 OD·mL/ng and LOD as low as 0.029 ng/mL for VHH detection. The cross-reactivity and recovery tests demonstrated the applicability of the assay to a broad range of VHHs with high precisions and minimal negative interference in the matrix. The real sample analysis reveals a significant loss of VHH proteins in the discarded supernatants that should draw more attention in the future. Although the VHH detected in the supernatant depends on the VHH sequence, expression system, and induction duration, the abundance of the VHHs remains at mg/ml levels in general. The highest amount of VHH detected in the supernatant samples reaches 41.3 mg/mL. The results of the study illustrated a high efficiency ELISA method for detection of varieties of VHHs in complex matrix. Modified versions of the reported sandwich ELISA could easily be applied to any steps during protein expression to determine the optimal expression condition and purification method for the production of VHHs.

## Supplementary Information

Below is the link to the electronic supplementary material.

Supplementary file1 (DOCX 35 kb)

## Data Availability

The datasets generated during and/or analyzed during the current study are available from the corresponding author on reasonable request.

## Abbreviations

VHH: Camelid heavy-chain single-domain antibody
pAb: Polyclonal antibodies
mAb: Monoclonal antibodies
ELISA: Enzyme-linked immunosorbent assay
sEH: Soluble epoxide hydrolase
PBS: Phosphate-buffered saline
BSA: Bovine serum albumin
SM: Skim milk
HRP: Horseradish-peroxidase
TMB: 3,3′,5,5′-Tetramethylbenzidine
*E. coli*: Escherichia coli
IPTG: Isopropyl-β-D-thiogalactopyranoside
OD 450: Optical density at 450 nm
LOD: Limit of detection
CV: Coefficient of variation
SD: Standard deviation
NSB: Nonspecific binding
HA: Hemagglutinin
3-PBA: 3-Phenoxybenzoic acid
AFB1: Aflatoxin B1
ATPE: Aqueous two-phase extraction
