# Supplementary material for: Development of a sandwich ELISA for the specific quantitation of hemagglutinin (HA)-tagged proteins during their inducible expression in Escherichia coli
Source: Anal Bioanal Chem. 2023 Jul 28;415(23):5563–74. doi: 10.1007/s00216-023-04846-w (PMC10473979; doi:10.1007/s00216-023-04846-w)
Supplement: Supplementary file 1 — Supplementary file1 (DOCX 35 kb) [file 216_2023_4846_MOESM1_ESM.docx]

**Supporting information for**

**Development of a sandwich ELISA for the specific quantitation of hemagglutinin (HA)-tagged proteins during their inducible expression in Escherichia coli**

Zihan Yin^a^, Qiyi He^a^, Huiyi Yang^a^, Christophe Morisseau^a^, El-Sayed A. El-Sheikh^c^, Dongyang Li^a,b^*, Bruce D. Hammock^a^*

^a^ Department of Entomology and Nematology, and UCD Comprehensive Cancer Center, University of California, Davis; California 95616; United States

^b^ Laboratory of Agricultural Information Intelligent Sensing, College of Biosystems Engineering and Food Science, Zhejiang University, Hangzhou, Zhejiang, 310058, China

^c^ Plant Protection Department, Faculty of Agriculture, Zagazig University, Zagazig 44511, Egypt

Corresponding Author: Bruce D. Hammock, bdhammock@ucdavis.edu

Dongyang Li, [dylee@zju.edu.cn](mailto:dylee@zju.edu.cn)

**Sample collection**

The recombinant DNA plasmids containing the VHHs and a 6×His tag at the C-terminus were available in our laboratory from previous works. The blank and supernatant samples were collected according to the same procedures, except blank plasmids (carries no protein insert) were used in the negative control. The plasmids were transformed into chemically competent cells of *E. coli* strain TOP10F’ using the heat shock method described as follow [1]. After a 30-minute incubation on ice, 1 μg/mL DNA plasmids were mixed with the TOP10F’ cells and placed in 42°C hot water bath for 90 seconds (heat shock) and then sit on ice for another 5 minutes. SOC media was added to the mixture and the transformed cells were incubated on shaker at 37°C for an hour. The cells were then cultured on super broth (SB) agar plates. The target positive colony obtained from the above step was amplified in SB media containing 50 μg/mL ampicillin (Amp) overnight and induced by 0.1 mM IPTG at 25 °C. One mL mixture of bacterial cells and the media was sampled two hours after the addition of IPTG and in the next day, respectively. The collected mixtures were centrifuged at 10,000g for 20 min and the supernatants from the centrifugation step were saved as the samples for ELISA analysis.

**Evaluation of Assay Reproducibility**

The reproducibility of the sandwich ELISA was evaluated by the plate-to-plate fluctuations. Plate I, II, and III were the plates run for assay optimization. The data were presented in **Fig. 2-4**. The sensitivity and LOD of these three plates under the optimal condition (i.e. 4 μg/mL capture antibody, 8.33 ng/mL (1:3000 dilution of the commercial product) HRP anti-HA mAb, and blocked by skim milk) were used for calculating the plate-to-plate average and CV. The results are presented in **Table S-1**.

**Table S-1** Summary of parameters in performance of the sandwich ELISA for VHH detection on three plates (n=3)

| **Plate** | **Slope/Sensitivity (OD mL/ng)** | **LOD  (ng/mL)** |
| --- | --- | --- |
| I (Figure 2) | 0.112 | 0.029 |
| II (Figure 3) | 0.090 | 0.044 |
| III (Figure 4) | 0.149 | 0.034 |
| **Mean** | 0.117 | 0.036 |
| **SD** | 0.029 | 0.007 |
| **CV (%)** | 25.2 | 20.8 |

**Reference**

1. Froger A, Hall JE (2007) Transformation of Plasmid DNA into E. Coli using the heat shock method. Journal of Visualized Experiments. https://doi.org/10.3791/253
